# High-Frequency Immersed Plasma: Reactive Species Generation, Redox Transformations, and Competing Chemical Processes in Iron-Induced Oxidative Degradation in a Deoxyribose Model System

**DOI:** 10.3390/ijms27104499

**Published:** 2026-05-18

**Authors:** Todor Bogdanov, Rene Mileva-Popova, Petar Iliev, Andrey Petrov, Plamena Marinova, Evgenia Benova, Nadya Hristova-Avakumova

**Affiliations:** 1 Department of Medical Physics and Biophysics, Faculty of Medicine, Medical University–Sofia, St. Georgi Sofiyski Str. No. 1, 1431 Sofia, Bulgaria; 2 Clean & Circle Center of Competence, Sofia University, 1a James Bourchier Blvd., 1164 Sofia, Bulgaria; 3 Department of Physiology and Pathophysiology, Faculty of Medicine, Medical University–Sofia, St. Georgi Sofiyski Str. No. 1, 1431 Sofia, Bulgaria; 4 Department of Pharmacology and Toxicology, Sector of Clinical Pharmacology and Therapeutics, Faculty of Medicine, Medical University–Sofia, St. Georgi Sofiyski Str. No. 1, 1431 Sofia, Bulgaria; 5 Faculty of Forest Industry, University of Forestry, 10 Kliment Ohridski Blvd, 1756 Sofia, Bulgaria

**Keywords:** cold plasma, plasma–liquid interaction, reactive oxygen and nitrogen species, oxidative degradation, deoxyribose assay, ferrous ions, redox cycling, Fenton reactions, hydrogen peroxide, plasma chemistry

## Abstract

High-frequency immersed plasma discharge represents an efficient method for the generation of reactive oxygen and nitrogen species (RONS) in liquid media, leading to complex redox and oxidative processes in biologically relevant systems. Although plasma-generated reactive species in liquids have been widely investigated, it remains insufficiently understood how working-gas-dependent plasma chemistry translates into oxidative outcomes in iron-containing model systems, where plasma-derived species may interact with transition-metal redox cycling. The novelty of this study lies in the combined assessment of gas-dependent RONS accumulation, deoxyribose oxidative degradation, and plasma-induced changes in Fe(II) availability using a high-frequency immersed plasma discharge. Herein, we examined whether treatment with high-frequency immersed discharge influences the redox state of iron in a working gas-dependent manner, thereby affecting oxidative degradation in the deoxyribose model. Plasma treatment was performed under air and argon working gas conditions, and oxidative degradation was evaluated using the thiobarbituric acid reactive substances (TBA-RS) assay. In parallel, the concentrations of long-lived reactive species, including hydrogen peroxide, nitrites, and nitrates, were determined spectrophotometrically. The results demonstrated a treatment-time-dependent increase in oxidative degradation and reactive species accumulation, with more pronounced oxidative effects observed under argon plasma conditions. In the presence of ferrous ions, plasma treatment resulted in a gas-dependent effect, characterized by a synergistic enhancement of oxidative degradation under argon and a biphasic effect under air. Most notably, in Fe(II)-containing samples, 10 min of argon plasma treatment increased TBA-RS formation to approximately 2.7-fold of the Fe(II) control, whereas air plasma produced a biphasic response, with an initial decrease followed by an approximately 40% increase at the longest exposure time. Additional experiments suggest that plasma may influence the redox state and availability of ferrous ions, thereby affecting their participation in Fenton-type reactions and radical-mediated processes. The findings suggest that the overall oxidative outcome in plasma-treated systems is governed not only by the concentration of plasma-generated reactive species but also by plasma-induced modifications of transition metal redox chemistry. These preliminary results on the combined roles of plasma-generated reactive species and transition-metal chemistry contribute to understanding plasma–liquid interactions in such systems.

## 1. Introduction

High-frequency immersed discharge is generated by gas ionization induced by a high electric field. It represents a non-equilibrium system consisting of charged particles, radicals, neutral, reactive species, and excited molecules, and emits electromagnetic radiation across the infrared (IR), visible (VIS), ultraviolet (UV), and vacuum ultraviolet (VUV) ranges, as well as producing transient electric and magnetic field fluctuations [1,2]. In several studies, it has been reported that gas discharge increases the concentration of reactive oxygen species (ROS) and reactive nitrogen species (RNS) in the exposed samples [3,4]. With respect to plasma-induced chemical and structural transformations of biomolecules, the crucial role is attributed to the combined action and remarkable diversity of these generated reactive oxygen and nitrogen species (RONS). In the present study, the term RONS includes both short-lived, highly reactive free radicals and long-lived oxidants with delayed chemical reactivity and characterized by the possibility of increased diffusion distances. Additionally, the complicity is enhanced by processes involving the transport of the generated species from the surrounding gas phase, through the plasma–liquid interface, and within the liquid phase of the suspending medium and multiple chemical reactions proceeding simultaneously or sequentially within the treated system [5,6]. The type and concentration of plasma-generated RONS can be modulated by changing the operational parameters used—power supply type, carrier gas composition, electrode configuration, applied voltage, treatment duration, gas flow rate, sample volume, and the distance between the electrode and the liquid surface [7,8]. Direct ROS/RNS-driven molecular oxidative modifications induced in a wide range of small organic molecules, peptides, lipids, and proteins have been extensively studied [9,10,11,12]. However, considerably less attention has been paid to how these gas-dependent differences translate into oxidative outcomes in simplified iron-containing model systems, in which plasma-derived species may interact with transition-metal redox chemistry.

In estimating free radical interactions, optical spectroscopic analyses have proven particularly suitable and frequently used. Among these methods, UV–VIS spectrophotometry, which measures the intensity of light absorbed by the sample solution, has been widely used because of its simplicity and reproducibility [13]. The assessment of oxidative interactions is accomplished by tracking the remaining unmodified probe molecules in the samples or by monitoring the modified or newly generated molecules formed as a consequence of oxidative interactions [14,15]. To estimate the oxidizing potential of the high-frequency discharge-generated mixtures of RONS, we searched for the possibility of quantifying oxidative damage degradation products using the TBA-reactive substances assay. The method is based on the reaction between malondialdehyde-like secondary end products of the oxidation process with two molecules of thiobarbituric acid (TBA). The formed pink colored complex could be subsequently spectrophotometrically determined at 532 nm. The reason to choose the TBA-RS assay is that it has proven adequate across different conditions of oxidative damage initiation. Both physical factors (UV, ionizing radiation, and thermal stress) and chemical substances (transition metals, such as ferrous iron or azo compounds, for example, 2,2′-Azobis (2-amidinopropane) dihydrochloride) could be used. These factors can be applied individually or in combination, allowing the assessment of their synergistic or modulatory effects. Moreover, the method has been used with a range of substances subjected to oxidative damage. During the years, simple one-component model systems containing a specific type of biologically important molecules (like deoxyribose solution) or multicomponent systems, i.e., lipid emulsions, biological fluids (plasma, serum, and cerebrospinal fluid), and tissue homogenates, have been used [16,17,18]. The method also enables evaluation of the effects of the tested substance or physical factors in both directions: suppression of oxidative damage (antioxidant activity) and enhancement of oxidative processes (pro-oxidant activity).

Despite the extensive literature on plasma-generated reactive species in liquids, less is known about how gas-dependent plasma chemistry influences oxidative outcomes in simplified iron-containing model systems, where plasma-derived species and iron redox reactions may occur simultaneously.

In the present study, we used a deoxyribose-based oxidative degradation assay to examine the effects of high-frequency immersed plasma treatment in the absence and presence of ferrous ions. We specifically compared air and argon as working gases in order to evaluate whether differences in plasma-generated long-lived reactive species are associated with differences in oxidative degradation and in the behavior of the iron-containing system. We hypothesized that, under otherwise comparable treatment conditions, the working gas composition would alter the balance of plasma-generated reactive species and thereby differentially affect deoxyribose oxidative degradation and Fe(II)-dependent oxidative processes. To address this, we quantified TBA-reactive products and selected long-lived reactive species, and we further examined whether plasma treatment influences the availability of Fe(II) in the tested system.

## 2. Results

The first step of our experimental work focused on identifying optimal treatment conditions for the selected model system to investigate the potential for oxidative damage resulting from exposure to plasma discharge. Two complementary experimental approaches were used. The first one comprised a 5-min high-frequency immersed discharge treatment of samples with different volumes (25 mL, 50 mL, and 75 mL). The second experimental series comprised treating samples with fixed volumes (50 mL) for 3 min, with a distinct pre-incubation period before the initial incubation step of the TBA-RS test. This experimental design enabled the simultaneous evaluation of the effects of several factors—the influence of sample volume, the potential impact of unavoidable delays during sequential irradiation on measurement outcomes, and the importance of exposure duration.

**Figure 1:**
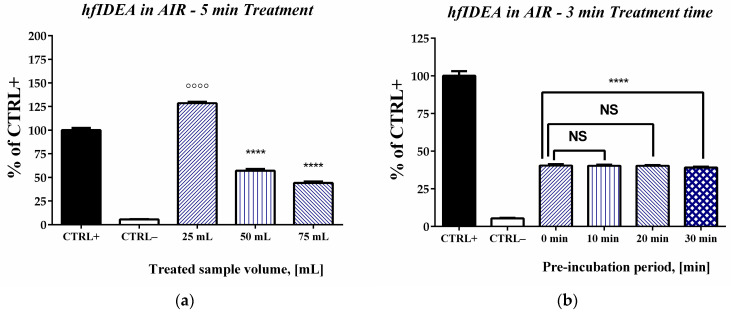
Preliminary study of the role of some experimental variables of plasma-induced deoxyribose degradation: (**a**) impact of plasma volume subjected to treatment; (**b**) effect of the pre-incubation period before the initial incubation step of the standard TBA-RS test. CTRL+—positive control containing 2-deoxy-D-ribose and standard chemical agents used for oxidative damage (Fe(II)—0.1 mM); CTRL−—negative control containing only the oxidizable substrate. Results are expressed as the mean ± standard deviation (*n* = 5). Statistical analysis was performed using one-way analysis of variance with Dunnett’s post hoc test. Significance was set at *p* < 0.05. Significantly higher or lower values compared to CTRL+ were marked with ᴼ and *, respectively (ᴼᴼᴼᴼ/**** *p* < 0.0001).

The experimental data from varying sample volumes, under conditions of a constant deoxyribose concentration in the reaction mixtures and identical discharge–source operating conditions, showed a statistically significant effect of the studied parameter (Figure 1a). The results from the three treated volumes differed significantly from those of the negative control (not treated with plasma discharge)—*p* < 0.0001. In the negative control, where no oxidative-damage-inducing chemical or physical factors were applied, we observed a negligible change in absorbance, corresponding to only 5.4% of the response measured in the positive control containing ferrous iron ions. For all tested volumes, the observed effect was several-fold greater than that recorded in the negative control. This increase in oxidative damage was volume-dependent. At the smallest volume used (25 mL), the estimated response reached 126.3% of the positive control, exceeding the level induced by the standard chemical agent. At the bigger volumes (50 mL and 75 mL), the measured absorbance values were lower than those of the positive control and close to each other. They corresponded to 54.6% for 50 mL and 41.9% for 75 mL. Based on the obtained data, a sample volume of 50 mL was selected for all subsequent experiments. At this volume, the response was significantly below the positive control, providing an adequate experimental range for evaluating the influence of treatment duration and other experimental parameters without approaching the system’s oxidative limits. The selected conditions also align with commonly applied optimization criteria for the assay and the principles of the Beer–Lambert law. The initial absorbance at 532 nm was adjusted to fall within the linear dynamic range (typically 0.2–0.5). Performing measurements within this interval minimizes signal saturation at higher absorbance values and avoids reduced sensitivity at lower levels. Maintaining control absorbance within this range ensures accurate, linear responses and enables reliable comparison of relative differences between samples under consistent experimental conditions.

The subsequent experiments aimed at optimizing the study conditions revealed that treatment time is also an important variable in our system (Figure 1b). The 3 min treatment of the 50 mL sample with gas discharge resulted in a statistically lower percentage of TBA-RS products compared to the positive control sample and the 5 min treated sample (Figure 1a). The values decreased to around 40%. These experiments also proved the lack of influence of the duration of the pre-incubation period if limited to no more than 20 min. Samples with no pre-incubation and pre-incubation for 10 and 20 min have statistically identical results, at around 40% of the positive control. Increasing the pre-incubation period to 30 min led to a slight but statistically significant decrease of 1%. For all subsequent experiments, the delay time before the initial incubation step of the standard TBA-RS test was fixed at 10 min, as this time duration was considered optimal with respect to the treatment time exposure and the other applied experimental procedures.

**Figure 2:**
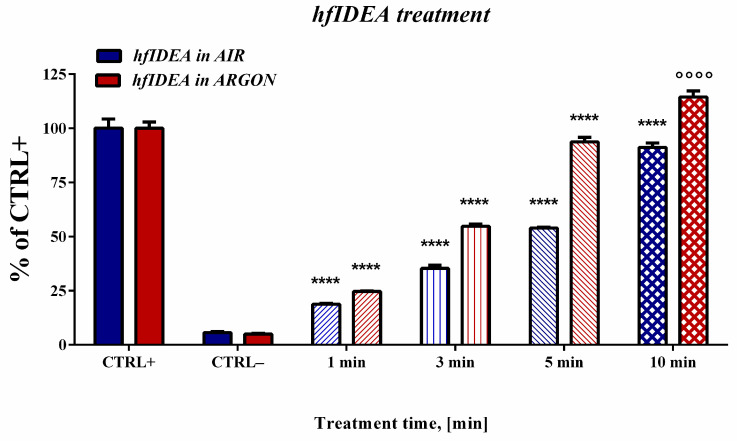
Effect of high-frequency discharge treatment time on deoxyribose degradation under AIR and ARGON flow conditions. Results are reported as the mean values ± SD (*n* = 5). Statistical comparison between the positive control (CTRL+) and other results was performed using one-way ANOVA (Dunnett’s post hoc test). Statistical significance was assumed when *p* < 0.05. Significantly higher or lower values compared to CTRL+ (CTRL+ Air in blue color; CTRL+ Argon in red color) were marked with ᴼ and *, respectively (ᴼᴼᴼᴼ/**** *p* < 0.0001).

The next step involved determining the influence of high-frequency discharge treatment time on deoxyribose oxidative degradation, comparing the response under AIR and ARGON flow conditions (Figure 2). As in the previous experiments, a positive control containing a standard chemical agent used to induce oxidative deoxyribose degradation (ferrous iron ions) and a negative control containing only untreated deoxyribose have been used. Regardless of whether air or argon gas flow was applied, a statistically significant increase in absorbance compared to the negative control was already observed at the minimum exposure duration of 1 min, indicating the generation of TBA-RS and deoxyribose degradation. In both working gases, a progressive increase in the absorbance and the relative TBA-RS levels was observed as the treatment time increased. Differences in the magnitude of this effect have been observed. Under air flow conditions at the first minute, the effect corresponded to less than one-fifth of the positive control (18.7%), whereas when argon gas flow was applied, the effect corresponded to one-quarter of the CTRL+ value (24.6%). This statistically significant increase in TBA-RS persisted throughout all subsequent treatment periods. The 5-min treatment with air, used as the plasma working gas, produced an effect comparable to that obtained after 3 min of argon exposure. At the maximal treatment duration in the case of air used as a working gas, the response approached the value of the positive control but did not reach the level induced by ferrous iron ions (91.2% of control). When using argon as the working gas, after 10 min of treatment, the measured value exceeded the response induced by the chemical agent by 15%.

**Figure 3:**
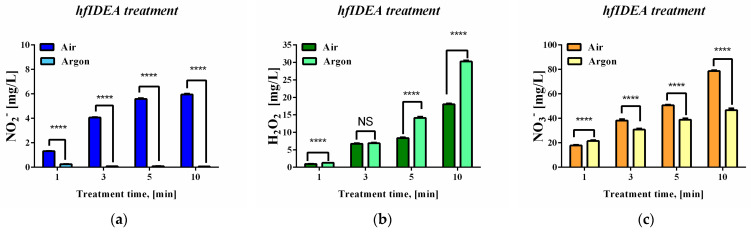
Long-lived reactive species concentrations during high-frequency immersed plasma treatment of a 3 mM deoxyribose solution in PBS buffer for different time intervals: (**a**) NO_2_^−^, (**b**) H_2_O_2_, and (**c**) NO_3_^−^. Results are reported as the mean values ± SD (*n* = 5). Statistical analysis was performed using two-way ANOVA followed by a Bonferroni post hoc test for multiple comparisons. Statistical significance was assumed when *p* < 0.05. (NS—not significant; **** *p* < 0.0001).

Figure 3 summarizes the concentrations of long-lived reactive species measured in the plasma-treated deoxyribose/PBS system in the absence of ferrous ions, in order to evaluate the gas-dependent plasma contribution under iron-free conditions. The corresponding iron-containing plasma-treated system is presented separately. The data presented in Figure 3 showed significant differences in the abundances of the different types of RONS under both operating gas conditions. When using air gas flow, the quantitative assessment of long-lived reactive species concentrations revealed increased RONS generation with increasing treatment time. The detected NO_2_– levels were lowest and remained below 10 mg/L even at the highest exposure time. Hydrogen peroxide levels surpassed this threshold after 10 min of exposure to plasma. Among the analyzed species, the NO_3_^−^ ion reached the highest concentrations in the model system suspending medium. A concentration of 15 mg/L was exceeded even after the extremely short treatment interval of 1 min. Differences between the working gases were also observed. The use of the inert mono-component working gas argon led to significant changes in the quantified NO_2_^−^ levels—a substantial reduction was recorded. A time-dependent increase in the amount of the two other RONS was again observed. A higher concentration of hydrogen peroxide was detected compared to that under air flow conditions. This effect was particularly pronounced at longer treatment times. Regarding NO_3_^−^, the detected levels were lower under argon gas flow, consistent with the observed changes in NO_2_^−^ levels.

Comparing the data presented in Figure 2 for the detected deoxyribose oxidative degradation and in Figure 3 concerning quantities of generated RONS, we observed a well-expressed trend of association between the increase in the sample solution of secondary products resulting from deoxyribose damage (TBA-reactive substances) and the accumulation of some of the resulting from the secondary reactions between the short-lived reactive species RONS. Linear regression reveals a strong linear association between TBA-RS and both H_2_O_2_ and NO_3_^−^, with coefficients of determination (R^2^) exceeding 0.98 under air gas flow. For argon gas flow regarding nitrate (NO_3_^−^), the strong linear trend is preserved, with the coefficient of determination (R^2^) remaining above 0.98. For hydrogen peroxide (H_2_O_2_), slight deviations from linearity are observed, resulting in a lower R^2^ value of approximately 0.88.

The observed trends suggest that the applied experimental design promotes radical-mediated oxidative processes within the system, together with the accumulation of long-lived reactive species formed through subsequent plasma–liquid and solution–phase reactions. The higher hydrogen peroxide concentration and the TBA-RS products observed under argon conditions led us to hypothesize that hydroxyl radical-mediated processes may substantially contribute.

In the next step, we applied a combined experimental approach to estimate deoxyribose degradation in the presence of both the chemical oxidizing agent and the plasma treatment. This approach enabled the evaluation of the discharge-induced effect of the standard oxidative damage initiated by ferrous iron ions. Experiments were again conducted under air and argon flow conditions. From the obtained data, it was apparent that both the magnitude and the overall response of both systems differed depending on the type of working gas used.

**Figure 4:**
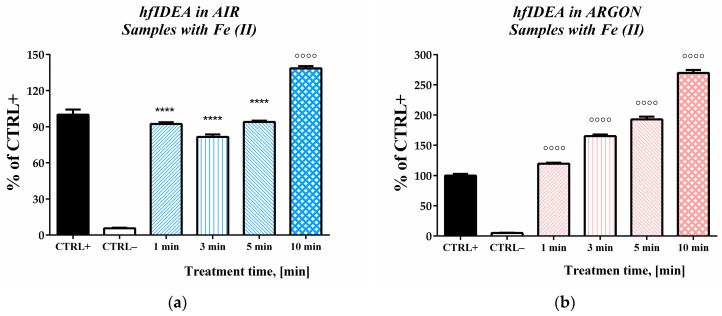
Effect of high-frequency discharge treatment time on deoxyribose degradation in the presence of ferrous iron under AIR (**a**) and ARGON (**b**) flow conditions. Data are reported as means ± SD (*n* = 5). Statistical comparison between CTRL+ and other results was performed using one-way ANOVA (Dunnett’s post hoc test). Statistical significance was assumed when *p* < 0.05. Significantly higher or lower values compared to CTRL+ were marked with ᴼ and *, respectively (ᴼᴼᴼᴼ/**** *p* < 0.0001).

In the model system, where argon was used as the working gas, simultaneous exposure to two factors inducing oxidative damage resulted in an accumulative increase in the extent of oxidative degradation and the generation of TBA-reactive substances (Figure 4b). This effect was observed across all tested treatment periods. Even in the shortest exposure time (1 min), the effect exceeded that of the iron-containing positive control by approximately 20%. At the maximal treatment time, it reached 2.7-fold higher levels. In the model system with gas discharge under air flow conditions, the obtained data did not indicate an association between treatment time and the number of TBA-reactive substances generated. The cumulative effect of increased TBA-RS products previously reported under the argon gas conditions was not observed at any of the tested exposure intervals. The obtained results revealed a biphasic relationship between exposure time and deoxyribose oxidative degradation, with variation across the investigated times, suggesting, at times, an opposite trend. The treatment of deoxyribose solution in the presence of ferrous iron ions with high-frequency immersed discharge under air gas flow for 1, 3, and 5 min did not result in an additive effect or an increase in the absorbance of these samples above that of the positive control to surpass the effect induced by the transition metal element. Instead, the calculated values, as a percentage of the positive control values, were significantly lower. The treatments with durations of 1 and 5 min had the same effect, with a more than 6% decrease compared to the CTRL+ sample. The lowest level of generation of TBA-reactive substances was observed in samples exposed to plasma for 3 min, with an approximately 20% decrease compared to the positive control. The higher value observed after 1 min of treatment compared to the 3 min result could be attributed to the domination of rapid ROS generation in the early treatment stages. With prolonged exposure, secondary reactions and redox transformations become dominant, resulting in a decrease in the observed response.

The decrease in the inhibitory effect on iron-induced deoxyribose degradation was no longer observed at the longest treatment duration (10 min). In these samples, we observed a pronounced increase in the number of TBA-reactive substances generated, and the effect exceeded that of the positive control by approximately 40%.

Another set of experiments was performed to quantify the long-lived reactive species in the PBS sample solution containing both the oxidizable substrate deoxyribose and the ferrous iron.

**Figure 5:**
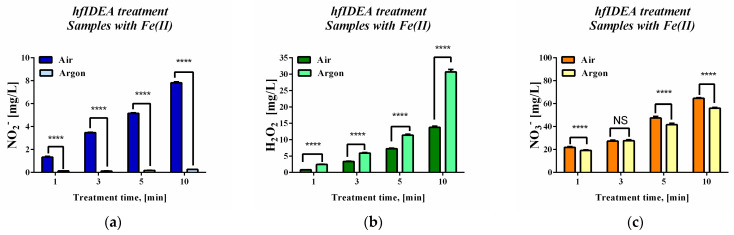
Long-lived reactive species concentrations during high-frequency immersed plasma treatment of a 3 mM deoxyribose solution in PBS buffer in the presence of ferrous iron for different time intervals: (**a**) NO_2_^−^, (**b**) H_2_O_2_, and (**c**) NO_3_^−^. Data are reported as the means ± SD (*n* = 5). Statistical analysis was performed using two-way ANOVA followed by a Bonferroni post hoc test for multiple comparisons. Statistical significance was assumed when *p* < 0.05. (NS—not significant; **** *p* < 0.0001).

The results presented in Figure 5 demonstrate that the presence of ferrous iron influenced the concentrations of long-lived reactive species detected after plasma treatment. Compared to the system without iron, some differences in the concentrations of hydrogen peroxide, nitrites, and nitrates were observed, indicating that additional chemical reactions involving iron ions occurred in the treated solution. Iron-catalyzed redox processes and Fenton-type reactions may also contribute to the consumption and transformation of some of the plasma-generated species. Nevertheless, the overall trends and relationships between the concentrations of the different species remained consistent. The type of working gas again significantly influenced the balance between different types of RONS. Under argon flow conditions, higher concentrations of hydrogen peroxide were detected, and under air flow conditions, higher concentrations of nitrogen-based species were measured.

These tendencies were in accordance with the synergistic effect observed under argon plasma treatment conditions, where the combined action of plasma-generated short and long-lived reactive species and ferrous ions led to enhanced oxidative degradation compared to the control. However, the measured concentrations of long-lived species indicating similarities in the pattern in the presence and absence of ferrous iron could not be associated with the biphasic effect observed under air plasma conditions. The unexpected results necessitated further analysis. We investigated the potential influence of thermal effects induced during plasma treatment, as well as the possible transition of the ferrous iron into another valence state unable to induce oxidative damage. In addition, potential plasma-induced changes in the pH of the treated solutions were experimentally evaluated, due to their known impact on iron chemistry and Fenton-like reactivity.

All these experiments were focused on the air plasma system, where the unexpected biphasic effect in the presence of iron ions necessitated further mechanistic analysis, whereas under argon plasma, the observed response followed the expected pattern.

First, we monitored the temperature variation of PBS deoxyribose solution in the presence and absence of ferrous iron under air conditions (Table 1). Heating of the samples was observed, with the extent of temperature increase varying depending on whether iron was present in the treated solutions. The magnitude of the effect increased with exposure time.

**Figure 6:**
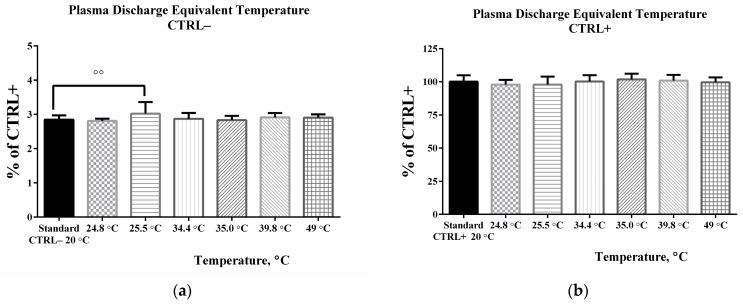
Impact of heating of control samples at a temperature equivalent to gas discharge induced: (**a**) negative control CTRL−; (**b**) positive control CTRL+; results are presented as the means ± SD (*n* = 5). Statistical comparison between CTRL+ and other results was performed using one-way ANOVA with Dunnett’s post hoc test. Statistical significance was set at *p* < 0.05. Significantly higher values compared to the controls at standard room temperature (CTRL+/CTRL− 20 °C) were marked with ᴼ (ᴼᴼ *p* < 0.01).

To quantitatively assess the potential contribution of this heating effect, experiments were performed with solutions whose chemical compositions, both qualitatively and quantitatively, corresponded to the positive and negative controls used in the discharge treatment experiments. Both types of controls have been exposed to identical temperatures for equivalent time intervals, with heating carried out gradually in a similar manner to gas discharge treatment experiments using a thermostatically controlled heating block (HB120-S, DLAB Science) (Figure 6). This approach enables fine temperature regulation and precise control of overheating conditions, facilitating close matching between the experimental setups, i.e., a gradual increase in the sample temperature but without fully representing the thermal environment at the plasma–liquid interface where localized heating may occur in the form of transient, spatially confined hotspots. From the results presented in Figure 6, it is evident that a statistical change was observed in only one of the negative controls, where the heating effect reached 25.5 °C. The negative control samples have extremely low absorbance values ranging from 0.018 to 0.025. Considering this fact and the absence of this effect or its further enhancement in other samples, it is most likely due to random variation. In all other reaction mixtures for the positive and negative controls, regardless of the magnitude of the thermal effect and the time duration of exposure to plasma, no statistically significant changes were observed. Although some of the tested temperatures surpassed the initial incubation temperature (37 °C), no statistically significant differences were detected, suggesting that bulk temperature increases for the applied time intervals alone may not fully explain the observed downstream effect.

pH was measured in all investigated solutions containing deoxyribose in PBS, both in the presence and absence of iron ions, after plasma treatment was performed. The initial buffer was adjusted to pH 7.40, and no significant deviations from this value were observed, regardless of the type of working gas used or the duration of the plasma exposure. At the maximal treatment time for air used as working gas, the pH was 7.34, and for argon, it was 7.41. This indicates that the buffering capacity remained stable.

**Figure 7:**
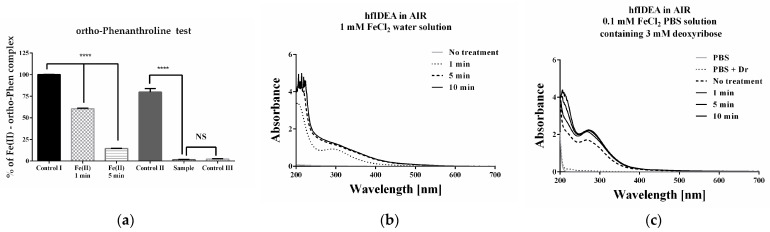
Assessment of available iron for chelation using the ortho-phenanthroline test: (**a**) Bar diagram showing the extent of formation of the Fe(II)-ortho phenanthroline complex. Three types of Controls have been used: Control I in distilled water; Control II containing FeCl_2_ dissolved in a 3 mM deoxyribose PBS solution; and Control III containing FeCl_3_. Data are presented from five independent experiments as the means ± SD (*n* = 5). Statistical comparison between Control I and other results was performed using one-way ANOVA (Dunnett’s post hoc test). Statistical significance was assumed when *p* < 0.05. Significantly lower values compared were marked with * (NS—not significant; **** *p* < 0.0001); (**b**) UV-Vis absorbance spectra of 1 mM water solution of FeCl_2_ treated with plasma under air flow conditions for different time intervals; (**c**) UV-Vis absorbance spectra of 1 mM PBS solution of FeCl_2_ containing 2-deoxy-D-ribose treated with gas discharge under air flow conditions for different time intervals.

With thermal effects or any significant changes in pH of the medium unlikely to be the primary contributing factor, the findings suggest that plasma alters the chemical state and modulates the redox cycling of ferrous ions. Therefore, as the next step, the influence of plasma discharge on the available ferrous iron for chelation was studied. An ortho-phenanthroline test was performed, and absorbance spectra of all solutions were taken. The experiments initially were conducted in distilled water—the preferred solvent. The discharge treatment resulted in significantly lower formation of the Fe(II)3-ortho-phenanthroline complex compared to Control I. The effect was dependent on the exposure time. After 1 and 5 min of treatment, the percentage of formation decreased to 60.61% and 14.52%, respectively (Figure 7a). The use of iron dissolved in PBS containing 3 mM deoxyribose, instead of distilled water, in Control II, resulted in a statistically significant decrease compared to Control I—likely phosphate interference. This effect was expected and reflects the influence of the PBS buffer on the methodology, which is observed even at a minimal amount (50 µL, 10 mM PBS). Under these experimental conditions, the fifth-minute plasma treatment under air gas flow conditions completely suppressed the complex formation, and the result was identical to control III, containing FeCl_3_, and no formation of the Fe(II)–orthophenanthroline complex was possible. This indicated that treatment affects the form of the available iron, which could be associated with variations in its capability to induce oxidative degradation of deoxyribose. The absorbance spectra taken, which showed well-expressed maxima between 250 and 350 nm, also confirmed this. They also excluded the possibility of generating products with significant absorbance at 532 nm. The last point indicates the lack of possibility of absorbance interference that could lead to misinterpretation of results.

The combined analysis of Figure 4, Figure 5 and Figure 7 demonstrates that the oxidative effects observed in the system were governed by a complex interplay between plasma-generated reactive species and iron redox reactions. The results indicated that not only the concentration of reactive species but also their interactions with transition-metal ions and the resulting radical chemistry determined the overall oxidative outcome of the plasma treatment.

## 3. Discussion

In the simplified model system treating PBS solutions of deoxyribose with high-frequency immersed discharge, we observed two well-defined, distinct trends. First, the extent of deoxyribose oxidative degradation increased with increasing treatment time, regardless of whether we used air or argon gas flow, and second, this effect was more pronounced under argon treatment conditions. These findings are consistent with the data on long-lived reactive species and oxidants (H_2_O_2_, NO_2_^−^, and NO_3_^−^), which clearly demonstrate that plasma-induced solution chemistry intensifies with prolonged plasma treatment.

The fact that the second effect, more pronounced oxidative damage of deoxyribose under argon gas flow, correlates with a higher concentration of H_2_O_2_, a minimal amount of NO_2_^−^, and a lower concentration of NO_3_^−^ draws our attention to the radical-mediated and secondary reaction pathways underlying both deoxyribose oxidative degradation and the formation of the detected long-lived reactive species, particularly the possible role of hydroxyl radicals as short-lived intermediates.

According to W. Boxem et al., who measured the concentrations of NO_2_^−^ and H_2_O_2_ in PBS treated with an argon plasma jet kINPen^®^, the hydrogen peroxide formation is likely associated with reactions involving O(^3^P) and O(^1^D) atoms in the plasma [19]. These oxygen atoms are mainly generated either due to electron-induced dissociation of molecular oxygen or through collisions between O_2_ and excited molecular nitrogen (Equations (1)–(6)) [20].

O_2_ + e^−^ → 2O(^3^P) + e^−^ (~6 eV) (1)

O_2_ + e^−^ → 2O(^1^D) + e^−^ (~9.97 eV) (2)

O_2_ + e^−^ → O(^3^P) + O(^1^D) + e^−^ (~8.4 eV) (3)

O(^3^P) + e^−^→ O(^1^D) + e^−^(~1.97 eV) (4)

O_2_ + N_2_* → 2O(^3^P) + N_2_ (5)

O(^3^P) + N_2_* → O(^1^D) + N_2_ (6)

Subsequently, the generated O(^3^P) and O(^1^D) can react with the solvent water molecules and generate hydroxyl radicals (Equations (7) and (8)).

O(^3^P) + H_2_O → 2^●^HO (unlikely and very slow) (7)

O(^1^D)+ H_2_O → 2^●^HO (main source) (8)

A minor contribution to hydroxyl radical formation may arise from the excitation of water molecules after a reaction with high-energy electrons or UV photolysis, near the plasma–liquid interface.

Under argon gas flow conditions, the inert gas can also participate in the generation of hydroxyl radicals by being transformed in an excited electronic state and subsequent interaction with water molecules (Equations (9)–(11)) [21].

e^−^ + Ar → Ar* + e^−^ (9)

Ar* + H_2_O → H_2_O* + Ar (10)

H_2_O* → ^●^HO + ^●^H (11)

The hydrogen peroxide detected in the liquid phase could be generated either in the gas phase via the reaction of the recombination of two hydroxyl radicals and subsequently transported in the sample solution (Equation (12)), or if the hydroxyl radicals manage to be transported intact to the liquid phase to perform a self-recombination (Equation (13)).

^●^HO_(gas)_ + ^●^HO_(gas)_ → H_2_O_2 (gas)_ → transition into the liquid phase → H_2_O_2 (liquid)_ (12)

^●^HO_(gas)_ → ^●^HO_(liquid)_ + ^●^HO_(liquid)_ → H_2_O_2 (liquid)_ (13)

Apart from the reaction outlined in Equation (13), additional competing reactions may occur, and the degree to which these reactions might happen is different depending on the gas flow conditions used during the treatment. Hydroxyl radicals exhibit extremely high reactivity, with a rate constant close to the diffusion limits 10^9^–10^10^ M^−1^ s^−1^. They have been proven to readily react with all organic molecules, including deoxyribose (k_2_DR = 3.1 × 10^9^ M^−1^ s^−1^). The interaction leads to the formation of five different deoxyribose radicals, although only one undergoes transformation into malondialdehyde-like compounds [22].

^●^HO_(gas)_ → ^●^HO_(liquid)_ + 2-DR_(liquid)_ → 2-DR_(liquid)_ – degradation → 2-DR^●^_(liquid)_ (14)

The fact that hydroxyl radicals are widely considered key intermediates in both the generation of H_2_O_2_ and deoxyribose oxidative damage draws attention, considering the possibility of secondary reactions that might also contribute.

Regarding differences in the extent of deoxyribose degradation depending on gas type, it should be noted that argon is an inert gas, while air is a complex multicomponent mixture composed of approximately 78% N_2_ and 21% O_2_. Under high-frequency immersed discharge conditions, a variety of short-lived RNS are formed. Two main pathways of their transformation are possible here. The first is the transformation to nitrite and nitrate, and the second is the transfer of NO and NO_2_ from the liquid to the gas phase [23]. The long-lived NO_3_^−^ and NO_2_^−^ are generated from nitrogen oxides, predominantly NO• and NO_2_• originating from gas-phase reactions between N_2_ and O_2_, where dissociation, excitation, and ionization processes occur. In this process of transformation among the different types of RNS, short-lived ROS such as ^●^OH are important intermediates.

In the case where argon is used as the working gas, however, the chemical reactions related to RNS pathways are significantly limited, as can be seen from our results indicating a lack of NO_2_^−^ and decreased levels of NO_3_^−^. They could proceed only due to the small amounts of N_2_ and O_2_ diffusing from the surrounding air into the discharge region. Consequently, fewer hydroxyl radicals would be used by these pathways, making them even more available for the main reactions responsible for deoxyribose degradation and H_2_O_2_ generation.

The data provided important insight—increasing treatment time clearly indicated that plasma discharge led to progressive accumulation of reactive species in the liquid phase. This observation was consistent with the time-dependent increase in deoxyribose degradation, which confirmed that the oxidative processes in the simplified model system were primarily driven by plasma-generated reactive species.

In the second part of our experiment, we used a more complex model system. Despite the fact that the only difference in the sample medium is the presence of FeCl_2_, the ability of ferrous ions to participate extensively in free radical processes, change valence, and engage in redox cycling processes substantially complicates the possible reaction pathways.

Here, we have an additional mechanism of deoxyribose degradation—apart from the plasma-induced ^●^OH, it can be oxidatively damaged because of ferrous ion autoxidation, the generation of diverse types of ROS, and direct interaction of Fe^3+^ with the biologically important molecule [24].

Fe^2+^ + O_2_ ↔ Fe^3+^ + O_2_^●–^ (15)

2O_2_^●–^ + 2H^+^ → H_2_O_2_ + O_2_ (16)

Fe^2+^ + H_2_O_2_ → Fe^3+^ + OH^–^ + ^●^OH (17)

^●^OH + 2-Deoxyribose → 2-Dr^●^ Decomposition (18)

Fe^3+^ + 2-Deoxyribose → Fe^3+^ − 2-Dr → Fe^2+^ + 2-Dr^●^ (19)

In the case of argon as the working gas, a synergistic effect between the chemical and physical initiating oxidative damage was observed. The resulting effect is not strictly additive, which could be explained by the occurrence of various parallel and competing chemical reactions in the system.

The effect in the presence of both initiating factors under air flow conditions also exceeded that observed under plasma when administered alone. Nevertheless, its biphasic pattern and lower response at shorter treatment intervals compared to the chemical agent, if used alone, indicate an alteration of ferrous iron’s capability to induce oxidative damage. Experiments investigating the possible contribution of plasma thermal effects showed that the biphasic effect observed during plasma treatment under air-flow conditions cannot be attributed to sample heating during the treatment. As indicated in the previous experiments (Figure 1 and Figure 3), the system’s maximal oxidative capacity was not reached. Therefore, if relevant, the presence of thermal effect would be expected to be associated with increased TBA-RS generation compared to the standard control samples. No evidence of such an effect was detected.

The experiments performed concerning the stability of the ferrous iron used for oxidative damage clearly proved that plasma treatment was associated with some changes, and the obtained product was dependent on the suspending medium used. The ortho-phenanthroline assay was applied to evaluate accessible Fe(II), while taking into consideration the fact that nitrite and phosphate-containing species may affect ferrous iron availability through redox and coordination processes [25,26]. Nitrite ions may oxidize Fe(II) to Fe(III). Under PBS conditions (pH 7.4), ferrous iron may coordinate with phosphate species and undergo oxidation to Fe(III), followed by hydrolysis and/or the formation of phosphate-associated Fe(III) complexes [27,28,29]. All of the mentioned reactions may affect the oxidative activity of the agent used to induce deoxyribose oxidative damage. In the water solution, the spectra of FeCl_2_ after treatment are characterized by the appearance of pronounced absorption bands observed around 200 and 300 nm. Comparison of the absorbance spectra with literature data suggests the formation of Fe(III) hydroxo complexes [30]. The results from the ortho-phenanthroline assay also indicate changes in the availability of Fe(II) in the PBS deoxyribose solution treated under air gas flow conditions. Given the fact that the buffering capacity of the system was preserved, no significant acidification is expected, and interactions involving the relatively low reactivity NO_3_^−^ ions are unlikely to play a major role. In contrast, for NO_2_^−^, which is only generated under air gas flow conditions and has a negligible concentration when argon is used as working gas, ions may participate in redox interactions with iron even under near-neutral conditions, potentially contributing to the observed changes in Fe(II) availability.

The higher degree of deoxyribose oxidative damage observed in samples treated with the chemical and physical factors compared to samples treated only with gas discharge under air flow conditions indicates a contribution of the presence of iron to deoxyribose degradation. The presence of oxygen and an abundant amount of oxidizing species accelerate Fe^2+^ oxidation. This participation of iron redox cycling, rather than contributing as a direct oxidizing agent for deoxyribose, could explain the decrease in TBA-reactive substance formation compared to the positive control containing iron.

Overall, in addition to radical generation, plasma treatment affected the chemical state and availability of ferrous ions, which altered their ability to participate in oxidative reactions. As a result, the overall oxidative effect observed in the system of deoxyribose oxidative damage induced by plasma under air gas flow and in the presence of ferrous ions was determined not only by the concentration of the generated RONS but also by plasma-induced modifications in iron redox chemistry.

## 4. Materials and Methods

### 4.1. Plasma Treatment

**Figure 8:**
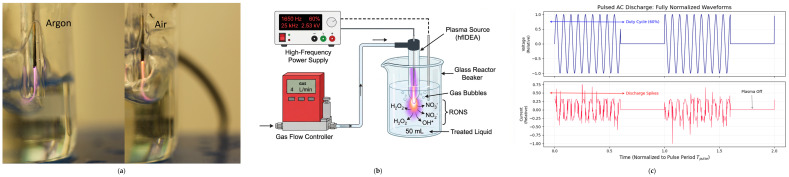
Experimental setup and electrical characterization of the high-frequency immersed plasma source: (**a**) photographic images of the discharge during operation in Ar and air; (**b**) schematic overview of the plasma treatment configuration and reactive species formation; (**c**) normalized applied voltage and discharge current waveforms over one pulse period.

Plasma treatment of the liquid samples was performed using a laboratory-developed high-frequency immersible discharge plasma source (hfIDEA), designed in our group for direct plasma treatment of liquids. The system is a laboratory-developed, non-commercial plasma source designed in our group and has not yet been described in a separate dedicated technical publication; therefore, the constructional and operating details relevant to the present study are provided here. Plasma is generated directly in the treated liquid by means of immersed electrodes connected to a high-frequency power supply, enabling an efficient plasma–liquid interaction and the formation of reactive oxygen and nitrogen species (RONS). A photographic image and a schematic overview of the experimental setup are shown in Figure 8.

The discharge assembly included centrally inside a quartz tube (8 mm outer diameter and 4 mm inner diametermultifilament copper electrodes with a cross-sectional area of 2.00 mm^2^. One of the electrodes was positioned ), while gas was introduced through a 3D-printed T-shaped connector providing a radial inlet flow and linking the electrode, gas supply, and glass discharge assembly. This configuration enabled stable gas delivery to the active discharge zone during liquid treatment.

The plasma source was operated with argon (99.995% purity) or air as the working gas at a flow rate of 4 L/min using a VÖGTLIN Red-Y Compact Thermal Mass Flow Meter (Vögtlin Instruments GmbH, Muttenz, Switzerland). The pulsed discharge was sustained with a pulse repetition frequency of 1650 Hz and a duty cycle of 60%. The high-frequency AC voltage had a run frequency of 25 kHz and an output voltage of 2.5 kV. The normalized applied voltage and discharge current profiles over one pulse period are shown in Figure 8c. These electrical parameters were measured under load, i.e., during operation with the discharge source immersed in the treated liquid. Under these operating conditions, gas discharge does not occur outside the liquid phase. The treated liquid volume in each experiment was 50 mL.

A direct calorimetric or Lissajous-based determination of the power deposited into the plasma/liquid system was not performed in the present study. Therefore, to avoid overinterpretation, only the experimentally measured under-load operating parameters are reported here.

The operating conditions have been selected to ensure stable discharge generation in the liquid and effective production of reactive species such as hydrogen peroxide, nitrates, nitrites, and other oxidizing agents responsible for the decontamination and physicochemical modification of the treated liquid.

### 4.2. Deoxyribose-Based Thiobarbituric Acid Reactive Substances Assay

The thiobarbituric acid reactive substances (TBA-RS) assay was employed to estimate the 2-deoxy-D-ribose degradation [31,32]. For this purpose, a solution of deoxyribose (3 mM) was prepared in phosphate-buffered saline (10 mM, pH 7.4). A physical factor (high-frequency immersed discharge using air or argon gas flow) and a chemical agent (FeCl_2_, 0.1 mM—autoxidation process) have been used for the initiation of deoxyribose degradation [33,34]. Although the FeCl_2_ concentration used in the positive control exceeds physiological levels, the deoxyribose assay is an extensively studied and frequently used model system for studying oxidative processes rather than replicating in vivo iron conditions [22,33,34]. The iron concentration used ensures sufficient radical generation. The obtained absorbance values were within the linear range of the TBA-RS assay, consistent with the Beer–Lambert law [24]. This enables accurate and reproducible quantitative comparisons. The described chemical and physical factors have been used separately and in combinations. The effect of increasing the plasma treatment duration was examined by applying treatments of 1, 3, 5, and 10 min. Four types of reaction mixtures were prepared:

The Positive control (CTRL+) was exposed only to the chemically induced oxidative damage and was not subjected to plasma treatment. The positive control contained deoxyribose 3 mM in PBS and FeCl_2_ 0.1 mM.

The Negative control (CTRL−) contained only deoxyribose 3 mM in PBS without being treated with chemical or physical factors initiating oxidative stress processes. In this control, no oxidative damage was expected during the chosen experimental conditions, and it was used to validate the method.

Sample type I was exposed only to the physical factors, contained deoxyribose (3 mM) in PBS, and was treated with plasma discharge for different time intervals under air or argon working gas conditions. It was used to evaluate the independent effect of plasma discharge and examine the potential impact of different gases on deoxyribose degradation.

Sample type II was exposed to both initiating factors, contained deoxyribose (3 mM) in PBS and ferrous iron (0.1 mM), and was treated with plasma discharge for different time intervals under air and argon working gas conditions. It was used to estimate the effect of gas discharge on iron-initiated deoxyribose degradation.

The first step of the assay comprised 30 min incubation of the prepared controls and samples at 37 °C. The next step was adding 0.5 mL of 2.8% trichloroacetic acid and 0.5 mL of 0.5% thiobarbituric acid. The mixture was vortexed and heated to 100 °C for 15 min. Samples were allowed to cool to room temperature, and the absorbance of the chromophore was measured at 532 nm. The results have been presented as a percentage of the positive control sample. No quenching agent was added at any stage of the assay since the experimental design aimed to evaluate deoxyribose oxidative degradation reactions occurring both during plasma treatment and during the subsequent incubation step.

### 4.3. Ortho-Phenanthroline Test

To investigate the impact of plasma on ferrous iron’s ability to maintain its second valence state and remain in the form capable of initiating oxidative damage, a test for the chelation of Fe(II) using ortho-phenanthroline was applied [35]. Two working solutions with equivalent concentrations of ferrous iron were prepared for treatment with high-frequency immersed discharge using air flow. The first used distilled water, and the second used a PBS solution of deoxyribose. Five types of reaction mixtures were prepared: Control type I—1 mL sample solution, containing 0.2 mM ortho-phenanthroline and 0.05 mM FeCl_2_; Sample type I—1 mL sample solution, containing 0.2 mM ortho-phenanthroline and 0.05 mM FeCl_2_ treated with plasma respectively for 1 and 5 min, respectively; Control type II—1 mL sample solution, containing 0.2 mM ortho-phenanthroline and 0.05 mM FeCl_2_ diluted in PBS solution of deoxyribose; Sample type II—1 mL sample solution, containing 0.2 mM ortho-phenanthroline and 0.05 mM FeCl_2_ diluted in PBS solution of deoxyribose treated with plasma for 5 min; Control type III—1 mL sample solution, containing 0.2 mM ortho-phenanthroline and 0.05 mM FeCl_3_. All reaction mixtures were incubated for 5 min to allow potential complex formation between the ferrous iron and the ligand. The absorbance was measured at 515 nm using a GENESYS 50 UV-VIS spectrophotometer to quantify the formation of the colored complex between ortho-phenanthroline and the available ferrous ions. The results have been presented as a percentage of the control type I. The absorbance spectra of all controls and samples, as well as individual components at the concentration used in the reaction mixtures, have been taken.

### 4.4. RONS Determination

**Figure 9:**
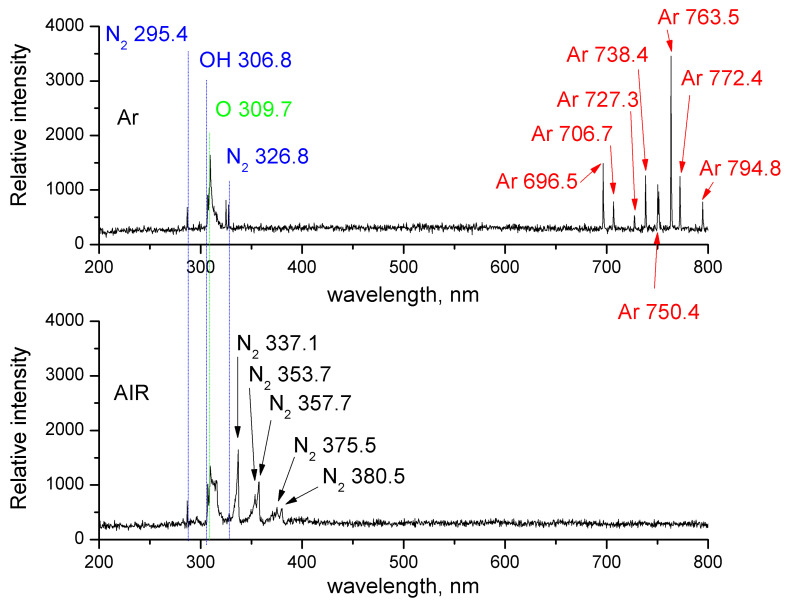
Optical emission spectra (OES) of the plasma source recorded during treatment of distilled water in argon (**upper panel**) and air (**lower panel**) in the 200–800 nm spectral range. Characteristic emissions assigned to OH, O, and N_2_ species, together with Ar emission lines in the argon discharge, are indicated, supporting the qualitative identification of excited RONS-related species generated by the plasma source.

The concentrations of selected long-lived reactive oxygen and nitrogen species (RONS), namely hydrogen peroxide (H_2_O_2_), nitrite ions (NO_2_^−^), and nitrate ions (NO_3_^−^), were determined using UV–VIS spectrophotometry. The analytical approach was based on colorimetric reactions previously applied for the characterization of plasma-treated liquids, including the methodology described by Čechová et al. [36], where hydrogen peroxide, nitrites, and nitrates in plasma-treated water were quantified spectrophotometrically using colorimetric reagents and UV–VIS detection. To complement the UV–VIS quantification of long-lived RONS in the liquid phase, optical emission spectroscopy (OES) was performed during plasma treatment of distilled water using argon and ambient air as working gases. The spectra were recorded with a Flame-S UV–VIS spectrometer (Ocean Optics B.V., Ostfildern, Germany) and are presented in Figure 9. The detected emission bands and lines, assigned mainly to OH, O, N_2_, and Ar species, provide qualitative evidence for the generation of excited RONS-related species by the plasma source and support the subsequent analysis of long-lived reactive species in the treated liquid.

Hydrogen peroxide concentrations were determined using a titanium reagent, Titanium(IV) oxysulfate–sulfuric acid solution, 27–31% H_2_SO_4_ basis, Sigma-Aldrich/Merck, Germany. The method is based on the reaction of hydrogen peroxide with titanium ions, resulting in the formation of a yellow peroxytitanyl complex. The absorbance of the formed complex was measured at 407 nm using a LAMBDA^®^ 365+ double-beam UV–VIS spectrophotometer (Perkinelmer Inc, Waltham, MA, USA). Hydrogen peroxide concentrations were calculated from a calibration curve prepared under the same experimental conditions.

Nitrite and nitrate concentrations were determined using commercial MQuant^®^ colorimetric test kits from Sigma-Aldrich/Merck, Germany: MQuant^®^ Nitrite Test Kit, colorimetric, 0.05–1.0 mg/L NO_2_^−^, Prod. No. 1.14658, and MQuant^®^ Nitrate Test Kit, colorimetric, 10–150 mg/L NO_3_^−^, Prod. No. 1.11169.0001. According to the manufacturer, these MQuant^®^ tests are intended primarily for visual colorimetric evaluation using a color card. In the present study, however, the tests were applied using an adapted spectrophotometric protocol: after the color reaction was developed according to the reagent procedure, the absorbance of the colored complexes was measured instrumentally instead of being evaluated visually by comparison with the color card.

For nitrite determination, the colored product was measured at 540 nm, while nitrate determination was performed at 380 nm, in accordance with the spectrophotometric wavelength selection reported for Sigma-Aldrich colorimetric reagents in plasma-treated water analysis by [36]. Calibration curves were prepared using standard solutions of the corresponding analytes and were processed under the same reaction and measurement conditions as the experimental samples. The concentrations of NO_2_^−^ and NO_3_^−^ were expressed in mg/L.

All measurements were performed after plasma treatment. For each experimental condition, the same adapted colorimetric–spectrophotometric protocol was applied in order to ensure comparability between samples treated under air and argon working gas conditions and between systems in the absence or presence of ferrous ions.

### 4.5. Statistical Analysis

Statistical analysis was performed using GraphPad Prism 8 (v8, GraphPad Software, La Jolla, CA, USA). The results from five independent measurements were reported as mean ± standard deviation. Statistical differences were determined using one-way ANOVA. Dunnett’s post hoc test was used when compared with the positive control (CTRL+), and Bonferroni’s correction was applied for multiple comparisons. Statistical significance was defined at *p* < 0.05 (ns—not significant; ᴼ/* *p* < 0.05; ᴼᴼ/** *p* < 0.01; ᴼᴼᴼ/*** *p* < 0.001; ᴼᴼᴼᴼ/**** *p* < 0.0001).

## 5. Conclusions

The present study demonstrates that the high-frequency immersed plasma source (hfIDEA) is an effective tool for generating reactive oxygen and nitrogen species in liquid systems and for inducing oxidative processes in a controlled model environment. Using a deoxyribose-based assay, we were able to evaluate both the direct oxidative effects of plasma-generated reactive species and their interaction with iron-induced oxidative mechanisms.

The results clearly showed that plasma treatment leads to time-dependent oxidative degradation of deoxyribose, accompanied by increasing concentrations of long-lived reactive species, such as hydrogen peroxide, nitrites, and nitrates. The type of working gas played a significant role in determining both the composition of the generated reactive species and the resulting oxidative effects. Argon plasma treatment resulted in higher oxidative degradation and higher hydrogen peroxide concentrations, consistent with a greater contribution of hydroxyl radical-mediated processes under these conditions. In contrast, air plasma treatment led to increased formation of nitrogen-based species and a different balance between reactive oxygen and nitrogen species, which influenced the overall oxidative outcome.

In more complex systems containing ferrous ions, plasma treatment demonstrated a dual effect depending on the gas composition. Under argon plasma conditions, a synergistic enhancement of oxidative degradation was observed, indicating the combined action of plasma-generated radicals and iron-catalyzed radical reactions. The most representative quantitative outcome was observed in Fe(II)-containing samples treated under argon, where 10 min of plasma exposure increased TBA-RS formation to approximately 2.7-fold of the Fe(II) control, emphasizing the interaction between argon plasma-generated reactive species and iron-mediated radical chemistry. Under air plasma conditions, however, a biphasic effect was observed, characterized by an initial decrease in iron-induced oxidative damage followed by an increase at longer treatment times. Additional experiments indicated that this effect was not caused by thermal factors but rather by plasma-induced changes in the chemical state and availability of ferrous ions. Spectrophotometric analysis suggested oxidation of Fe(II).

Overall, the obtained results indicate that the oxidative effects observed in plasma-treated liquid systems depend on a complex interplay between plasma-generated reactive species, solution chemistry, and transition-metal redox processes. Within the limits of the applied deoxyribose model, the study shows that plasma treatment may influence not only the accumulation of reactive species but also the availability of ferrous ions and the resulting oxidative response. These findings provide a basis for further investigation of gas-dependent plasma effects and iron-related redox transformations in more complex chemical and biological systems.

## Figures and Tables

**Table 1:** Temperature changes in PBS (10 mM, pH 7.4) containing 2-deoxy-D-ribose in the presence or absence of ferrous iron during high-frequency immersed discharge treatment under air flow conditions.

Exposure Time Duration	10 mM PBS Buffer with Deoxyribose [3 mM]	10 mM PBS Buffer with Deoxyribose [3 mM] in the Presence of Ferrous Iron
0 min	20.0 °C	20.0 °C
1 min	25.5 °C	24.8 °C
5 min	34.4 °C	35.0 °C
10 min	49.0 °C	39.8 °C

## Data Availability

The original contributions presented in this study are included in the article. Further inquiries can be directed to the corresponding author.

## Abbreviations

The following abbreviations are used in this manuscript:

hfIDEA	high-frequency Immersible Discharge for Environmental Applications
ROS	Reactive oxygen species
NOS	Reactive nitrogen species
RONS	Reactive oxygen and nitrogen species
UV-VIS	Ultraviolet–visible light region
TBA	Thiobarbituric acid
TBA-RS	Thiobarbituric acid reactive substances
PBS	Phosphate-Buffered Saline
CTRL+	Positive control
CTRL−	Negative control
OES	Optical emission spectra
